# Schizotypal Traits in Children with Autism Spectrum Disorder and the Impact on Social, Emotional and Behavioral Functioning

**DOI:** 10.3390/brainsci15070668

**Published:** 2025-06-20

**Authors:** Evdokia Tagkouli, Evangelia Chrysanthi Kouklari, Bruce J. Tonge, Vassiliki Ntre, Artemios Pehlivanidis, Nikos C. Stefanis, Christos Pantelis, Katerina Papanikolaou

**Affiliations:** 1 Department of Child Psychiatry, Aghia Sophia Children’s Hospital, National and Kapodistrian University of Athens, 115 27 Athens, Greece; etagkouli@gmail.com (E.T.); evakouklar@gmail.com (E.C.K.); 2 Centre for Developmental Psychiatry and Psychology, Department of Psychiatry, Monash University, Clayton, VIC 3168, Australia; bruce.tonge@monash.edu; 3 Aghia Sophia Children’s Hospital, 115 27 Athens, Greece; vassilikintre@yahoo.gr; 4 1st Department of Psychiatry, National and Kapodistrian University of Athens, Eginition Hospital, 115 28 Athens, Greece; apechlib@med.uoa.gr (A.P.); nistefan@med.uoa.gr (N.C.S.); 5 Department of Psychiatry, The University of Melbourne, Parkville, VIC 3010, Australia; cpant@unimelb.edu.au; 6 Florey Institute of Neuroscience and Mental Health, The University of Melbourne, Parkville, VIC 3010, Australia; 7 Monash Institute of Pharmaceutical Sciences (MIPS), Monash University, Royal Parade, Parkville, VIC 3010, Australia

**Keywords:** autism spectrum disorder, schizotypy, social functioning, emotional functioning, behavioral functioning

## Abstract

**Background:** Schizotypal traits are considered to be clinical and cognitive features of Schizotypal Disorder in children (SDc). These traits are also seen in children and adolescents with high-functioning Autism Spectrum Disorder (ASD). This study examines the influence of schizotypal traits (and their severity) on the capacity of children with ASD to manage emotions, develop relationships with others, and adapt in school and family life. **Methods:** The Schizotypal traits of 63 children (6–12 years old) with High Functioning ASD were measured by the Melbourne Assessment of Schizotypy in Kids (MASK). Parents and teachers of the participating children completed the Child Behavior Checklist (CBCL) and Teachers’ Report Form (TRF) from the Achenbach System of Empirically Based Assessment and the Aberrant Behavior Checklist (ABC). **Results:** Overall, the results indicated correlations between the MASK scores and problems recorded by teachers, such as Internalizing problems (i.e., Anxious/Depressed, Withdrawn/Depressed, and Other problems score) according to TRF and Inappropriate speech scores, according to teacher’s ABC scales. Schizotypal traits impact the social, emotional, and behavioral functioning of children with ASD at home and school environments. **Conclusions:** The assessment of schizotypal traits in children with ASD provides critical information about a child’s functionality and cognitive development, also leading to the identification of potential cognitive-neuropsychological endophenotypes within ASD with characteristics of both Autism and Schizophrenia spectra. Τhe development of a valid assessment tool is required, as well as the design of targeted interventions to prevent the loss of functionality.

## 1. Introduction

The term Autism Spectrum Disorder (ASD) describes a unique group of neuropsychiatric disorders characterized by *deviations and delays* in the development of specific skills beginning in the early years of life. The modern classification systems, DSM-5 of the American Psychiatric Association [1] and ICD-11 of the World Health Organization [2], agree that the characteristic-diagnostic behaviors for ASD are: (1) the presence of deficits in social communication and social interaction and (2) restricted, repetitive patterns of behavior, interests, and activities, even if other conditions may be present such as motor coordination disorder, language disorder, specific learning disorder.

Schizotypy as a concept describes a continuum of personality characteristics, ‘schizotypal traits’ with a factor structure consisting of three identifiable factors: ‘cognitive-perceptual,’ ‘interpersonal,’ and ‘disorganized’ [3,4]. These factors correspond to the ‘positive,’ ‘negative,’ and ‘disorganized’ dimensions of schizophrenia [5,6]. However, Stefanis et al. [7] proposed a four-factor model in which ‘positive schizotypal’ traits were further divided into a ‘paranoid’ factor and a ‘cognitive/perceptual’ factor. The four-factor model seeks to provide a more refined and comprehensive differentiation of schizotypal traits.

The present study is based on the Melbourne Assessment of Schizotypy in Kids (MASK), a semi-structured assessment developed to evaluate features of Schizotypal Personality Disorder (SPD) in children aged 5 to 12 years. Factor analysis of the MASK identified two distinct MASK factors: ‘Social/pragmatic’ and ‘Positive schizotypal’ [8].

Schizotypal traits are considered to be clinical and cognitive features of Schizotypal Disorder in children (SDc), which is categorized within the schizophrenia spectrum. Some of these traits, particularly difficulties in social cognition, atypical communication styles, and rigid or eccentric behavioral patterns, have been observed in children and adolescents with high-functioning Autism Spectrum Disorder (ASD) (e.g., Abu-Akel et al., 2015 [9]). However, while both conditions involve social difficulties, the underlying mechanisms differ; for instance, ASD is primarily characterized by deficits in social reciprocity and pragmatic language, whereas schizotypal traits may involve cognitive-perceptual distortions, such as magical thinking or unusual beliefs, which are not core features of ASD (American Psychiatric Association, 2013 [1]). Given this distinction, further research is needed to explore potential areas of overlap and differentiation between these conditions.

The phenotypic expressions of both autism and schizophrenia spectrum disorders are thought to span a continuum, ranging from mild subclinical levels in the general population to severe clinical manifestations [10]. The present study is designed based on the model of *the fully dimensional approach* outlined by Claridge and colleagues, which suggests that schizotypy (*schizotypal traits*) is on a continuum applying to all members of the population [5]. It views schizotypy as a spectrum of ‘natural central nervous system variations’, which in their extreme manifest as vulnerability to mental illness [5].

### 1.1. Psychiatric Comorbidity of Autism Spectrum Disorder

According to the US Centers for Disease Control and Prevention’s Autism and Developmental Disabilities Monitoring (ADDM) Network [11], the prevalence of ASD for the year 2022 is estimated at 1 in 31 children aged 8 years. The most recent estimated prevalence is 4.8 times higher than the first ADDM survey conducted 22 years ago in 2000. ASD is diagnosed more frequently in men than women in all age groups [12,13]. The estimated prevalence of ASD in Greece is 1.15% with males/females ratio, 4.14:1 and an approximate mean age at diagnosis of six years and one month [14].

ASD is an extremely heterogeneous category in terms of the severity of behaviors but also in the range of comorbid neurodevelopmental, organic, and psychiatric conditions [15,16]. In recent years, the issue of psychiatric comorbidity in ASD has gained increasing relevance as the DSM-5 now allows additional diagnoses in individuals with ASD [17].

Simonoff et al. [18], in one of the major epidemiological studies in ASD, recorded one comorbid disorder in 77% of the study group and two or more in 41% of that group, where 29% includes social phobia, 28% attention-deficit/hyperactivity disorder (ADHD), 28% Oppositional Defiant Disorder and 1.4% depression-dysthymia. Hossain et al. [19], in a systematic search (*umbrella review*), revealed a high rate of comorbid psychiatric disorders, including anxiety, depressive, bipolar, and mood disorders; schizophrenia spectrum; suicidal behavior disorders; ADHD; and disruptive, impulse-control, and conduct disorders. The prevalence of schizophrenia spectrum and other psychotic disorders was inconsistent, with differences ranging from 4 to 67%. Zheng et al. [20], in a systematic review and meta-analysis, demonstrated that the prevalence of schizophrenia was significantly higher in individuals with ASD than in controls, and the prevalence of ASD in individuals with schizophrenia ranged from 3.4 to 52%.

### 1.2. Association Between Autism Spectrum Disorder and Schizophrenia Spectrum Disorder

Although schizophrenia and autism are now regarded as two distinct conditions, they share a common history. Early assumptions of continuity between severe childhood psychiatric disturbance and adult schizophrenia were based on the severity of the two disorders. Autism was initially described as infantile psychosis and for a long time was approached as a form of schizophrenia until Kolvin in the year 1971 in the UK was able to separate the two conditions and record their differences [21]. According to the DSM-5, the Schizophrenia Spectrum and Other Psychotic Disorders are defined by symptoms in at least two of the following five domains: delusions, hallucinations, disorganized thinking (speech), grossly disorganized or catatonic behavior and negative symptoms. The negative symptom domain consists of five key constructs: blunted affect (reduced range of emotions, lacking facial expression), alogia (reduction in quantity of words spoken), avolition (reduced goal-directed activity due to decreased motivation), asociality (social withdrawal), and anhedonia (reduced experience or expression of pleasure) [22].

Autism and Schizophrenia spectra resemble each other. With regard to the negative symptoms, many adults with ASD show symptoms such as lack of interest in socializing, flat and restricted affect, inability to show emotions, restriction of facial expressions, diminished eye contact, poor speech, and slow movement. Also, some individuals with high-functioning ASD sometimes have difficulties that resemble the positive symptoms, such as thought disorder (i.e., poor reality testing, cognitive slippage, and perceptual distortions) [23,24]. However, children with ASD can develop psychotic symptoms in adolescence [25].

Both Autism and Schizophrenia Spectrum conditions involve complex developmental models manifested by deficits in social interaction, communication, executive function, and sensorimotor gating [26,27]. Both are extremely heterogeneous and polygenic and are considered among the most heritable disorders in Psychiatry (50–80% heritability) [28,29]. However, high rates of heritability have been reported among these disorders. Specifically, elevated rates of Autism Spectrum Disorder (ASD) have been observed in children of parents with Schizophrenia, increased prevalence of ASD traits has been found in siblings of individuals with early-onset Schizophrenia, and higher rates of Schizophrenia spectrum disorders have been documented in parents of children with ASD [30]. Studies of Copy number variants (CNVs) and other rare alleles have shown overlap between autism and schizophrenia and suggest that they may share underlying pathogenic mechanisms [22]. Also, both have been extensively associated with impairments in functional brain connectivity, and brain neuroimaging studies show corresponding correlations between the two disorders. Finally, for both conditions, shared genetic and environmental risk factors have been identified [31].

### 1.3. Children with Autism Spectrum Disorder and Schizotypal Traits

Although the “true schizophrenia” occurring in the context of ASD is uncommon, a subgroup of individuals within ASD may be at an increased risk of developing psychotic disorders [15]. It is established from the modern literature as well as from the clinical practice that children and adolescents with a diagnosis of ASD may present traits or even meet the criteria for the diagnosis of Schizotypal personality disorder or Schizotypal disorder in children (SDc). The incidences of schizophrenia spectrum were significantly higher than the comparison group across all three subtypes of autism-spectrum disorder [32]. Schizotypal disorder is categorized within the Schizophrenia Spectrum Disorders and its manifestation is recognized as early as childhood. Diagnostic criteria of schizotypal disorder according to the DSM-5 consist of a pervasive pattern of social and interpersonal deficits such as bizarre fantasies (magical thinking) and preoccupations, οdd behavior, odd thinking and speech (e.g., vague, casual, metaphorical, over-elaborated, or stereotyped), unusual perceptual experiences, paranoid ideation, disturbed affect, ideas of reference, difficulty making and keeping friends, anxiety, and mood disturbances [33,34].

Regarding the influence of Early-onset schizophrenia (EOS) on the level of children’s functionality, it is already known from the literature that it is one of the most devastating psychiatric disorders with a serious impact on development, more pronounced early neurodevelopmental abnormalities, and poorer prognosis and treatment response [35].

There are many different reasons for children and adolescents with autism and psychosis to become socially and behaviorally disturbed. In ASD, difficulties have been observed with social communication and interaction, characterized by a tendency to engage in repetitive, stereotyped patterns of immature/unusual behaviors. These problems are likely the result of a rigid thinking style and problems with social cognition, empathy, motor coordination, and sensory processing [28]. In SPD, which is included within the schizophrenia spectrum, social interaction deficits and solitary tendencies have been recorded, which are related to thought disorder, magical thinking, odd speech and ideation, and preoccupations with bizarre fantasies and interests [8]. Both situations can be at risk in psychosocial terms. Further, these children do not have the skills they need to cope in a new environment like a school, and they fail to face difficulties and challenges, resulting in their being judged and discriminated against by others [36].

Τhere has been relatively little research on how schizotypal traits affect the functioning of children with Autism Spectrum Disorder. Abu-Akel et al. [37] studied the impact of comorbid autism and schizotypal disorder in children on socio-pragmatic skills and executive functioning (i.e., intra-/extra–dimensional set-shifting task) in a group of a total 67 (6–12-year-old) children, 15 with autism, 8 with schizotypal disorder, and 12 with comorbid autism and schizotypal disorder, as well as 32 typically developing children. According to the results, the performance of the comorbid group (dual diagnosis) on socio-pragmatic skills was superior to the groups diagnosed with autism and schizotypal disorder separately. This could indicate the existence of some kind of cognitive mechanism that works in a compensatory manner when autism and schizophrenia spectra coexist [37].

### 1.4. Current Objectives

Functional impairment is a defining characteristic of both autism spectrum disorder (ASD) and schizophrenia spectrum disorders (SSD), as outlined in the DSM-5 and ICD-11. However, the relationship between these conditions within individuals and the potential impact of their co-occurrence on functional outcomes remains unclear.

Previous studies [37,38], have explored various models to understand the joint effects of autistic and psychotic symptoms, yielding contrasting results that range from functional benefits to adverse effects. Recent evidence indicates that the functional benefits of co-occurring ASD traits and positive SSD symptoms may depend on the severity of the symptoms [39].

The aim of the current study is to investigate the impact of such co-occurrence on social, emotional, and behavioral functioning by studying children diagnosed with ASD who also exhibit features of SSD, such as the schizotypal traits, of different severity (that means even when the criteria for SPD diagnosis are not met).

## 2. Materials and Methods

### 2.1. Participants

The research sample included 63 children between 6 and 12 years old with Autism Spectrum Disorder in accordance with the diagnostic criteria for ASD in DSM-5 (American Psychiatric Association, 2013), with or without schizotypal characteristics, consecutively coming for assessment (initial evaluation or follow–up) to the outpatient Autism Spectrum Disorders (ASD) clinic for children at the Department of Child Psychiatry at a university-affiliated children’s hospital in a major city during the years 2017 to 2020.

### 2.2. Procedure

This study was approved by the Institutional Review Board (IRB) of Aghia Sophia Children’s Hospital, in accordance with the ethical guidelines set forth by the Declaration of Helsinki and national regulations for research involving human participants (Approval No: 714/10/1/2018).

Parents of children fulfilling the study criteria were fully informed on the research procedure and had to submit written informed consent. In addition, age-appropriate assent was obtained from the children to ensure their voluntary participation. Thereafter a thorough social, developmental, and psychological history was taken from candidates’ families. Demographic and socioeconomic status were also registered in a special “registration form” created by the researcher. Full anonymity and privacy were secured during the registration of the results.

Initially, the children were assessed by a child and adolescent psychiatrist in order to ensure Autism Spectrum Disorder diagnosis according to the diagnostic criteria of DSM-5. In addition, the Greek version of the Wechsler Intelligence Scale for Children-3rd edition—WISC-III was administered by a psychologist along with a battery of diagnostic tools and questionnaires in Greek as described below.

The inclusion criteria for the study were as follows: Participants were required to have a diagnosis of Autism Spectrum Disorder, with severity levels categorized as either level 1 (“requiring support”) or level 2 (“requiring substantial support”). These levels reflect the degree of support needed in daily life and the extent to which autistic traits differ from neurotypical expectations. Additionally, participants needed to have a Full Scale Intelligence Quotient (FSIQ) score of more than 70, based on the WISC-III assessment. Exclusion criteria included a diagnosis of any other neuropsychiatric disorder, such as ADHD, or neurological conditions, such as epilepsy. Furthermore, participants could not be taking any medications, such as psychotropic or antiepileptic drugs, that might affect cognition. Finally, a good knowledge of the Greek language was essential to ensure participants could understand and provide reliable responses to interviews and questionnaires.

### 2.3. Measures

#### Clinical Tools

For the purpose of the research, a battery of diagnostic tools and questionnaires in Greek are given to children, parents, and teachers, described below:

**WISC**-**III (Wechsler Intelligence Scale for Children-third edition—Wechsler, 1992).** The WISC-III is considered the most valid and reliable psychometric tool for assessing the intellectual ability of children and adolescents aged between 6 and 16 years old. It comprises two scales, Verbal and Performance (non-verbal). Each scale contains subtests. The Verbal subtests are: Vocabulary, Similarities, Arithmetic, Information, and Comprehension. The Performance subtests are: Object, Assembly, Coding, Block design, Picture arrangement, and Picture completion. The WISC provides standard scores on Verbal IQ, Performance IQ and Full scale IQ [40].

**The Melbourne Assessment of Schizotypy in Kids—MASK (Jones et al., 2015 [8]).** The MASK is a semi-structured assessment that was developed to measure Schizotypal Personality Disorder (SPD) features in children between 5 and 12 years old [8]. The MASK collects information on symptomatology from three data sources: the child, the caregiver, and the assessing clinician. It comprises three components: Background Interview, Child Clinical Interview, and Clinical Presentation Checklist.

The *Background Interview* is conducted with the parent of the child and provides information about the child’s schizotypal symptoms.

The *Child Interview* is semi-structured and consists of questions that explore features of childhood SPD. This type of assessment helps children who may be anxious or embarrassed about their thoughts or have pragmatic difficulties interfering with language comprehension. The *Clinical Presentation Checklist* includes 57 observable features of childhood SPD that are assembled within nine domains: Social anxiety, Social skills, Motor abilities, Language/Thought/Ideation, Fantasy/Magical thinking, Unusual perceptual experiences, Behavior, Attention and Affect. Each item is rated on a Likert scale (Never, Sometimes, Often, and Always) by the child’s clinician only after obtaining information from both the child and the parent(s).

Jones et al. [8] noted that MASK was shown to have psychometric properties with high internal consistency (Cronbach alpha coefficient for the 57 MASK items was 0.98. Alpha coefficients for the nine subscales of the MASK were as follows: social anxiety (0.86); social skills (0.94); motor abilities (0.92); language/thought/ideation (0.93); fantasy/magical thinking (0.86); unusual perceptual experiences (0.85); behavior (0.88); attention (0.93); and affect (0.73)) and high inter-rater reliability (0.98). Finally, it is noted that the Total MASK score was excellent at distinguishing SPD as described in accordance with the criteria in DSM-5. A factor analysis revealed two MASK factors: Social/pragmatic and Positive schizotypal (Jones et al., 2015 [8]). Social/pragmatic factor and Positive schizotypal factor were associated with SPD, but only the Social/pragmatic factor was associated with ASD. The results showed high internal consistency for the MASK, which can provide a reliable measure of schizotypal symptoms across groups of SPD, ASD, and typically developing children. Also, Jones et al. (2015) [8] noted satisfactory convergent validity between the MASK and behavior rating scales: The Behavioral Assessment System for Children-Second Edition (BASC-II) [41] and The Conner’s Rating Scale-Revised (CRS-R) [42].

In the present study, all Cronbach’s α reliability coefficients were above the acceptable limit (0.7), indicating acceptable reliability of the MASK scale. Cronbach alpha coefficient for the 57 MASK items was 0.95. Alpha coefficients for the nine subscales of the MASK were as follows: social anxiety (0.78); social skills (0.86); motor abilities (0.95); language/thought/ideation (0.88); fantasy/magical thinking (0.93); unusual perceptual experiences (0.85); behavior (0.70); attention (0.72); and affect (0.78).

### 2.4. Behavior Rating Scales

**Achenbach System of Empirically Based Assessment—ASEBA/school-age assessment forms: 6–18 years old (Achenbach, 2001 [43]).** The Achenbach System of Empirically Based Assessment (ASEBA) is a comprehensive system of instruments for assessing abilities, adaptive functioning, and behavioral, emotional, social, and thought problems of people of various ages (from age 1½ to 90+ years). Achenbach’s questionnaires are universally accepted and used widely for children and adolescents. The purpose of using them was to assess the children’s strengths and problems in diverse environments and obtain information from the different sources (parents and teachers).

For the purpose of our research, school-age forms for children aged 6 to 18 were utilized, including the Caregiver Report Form, the Child Behavior Checklist (CBCL/6–18), and the Teacher’s Report Form (TRF/6–18) [43].

Each questionnaire consists of two parts. The first contains a series of questions assessing adaptive behavior, forming three scales on the CBCL—Activities, Social competence, and School competence—and five on the TRF—Academic performance, Working hard, Behaving appropriately, Earning, and Happy.

The second part of the CBCL and TRF consists of 112 and 113 items, respectively, which describe various aspects of the child’s behavior (at the time of assessment or within the past 6 months) rated on a 3-point scale (0—Not True, 1—Somewhat/Sometimes True, 2—Very True/Often True). These ratings are combined to form eight narrowband subscales or syndromes, two broadband scales, and a total problem score. The eight syndromes (*empirically based syndrome scales*) are based on factor analyses and labeled—Withdrawn, Somatic complaints, Anxious/Depressed, Social problem, Thought problem, Attention problem, Aggressive behavior, and Delinquent behavior. The broadband scales are termed Internalizing and Externalizing. The Internalizing problems scale is composed of Withdrawn, Somatic complains and Anxious/Depressed subscales. The Externalizing problems scale is composed of Aggressive behavior and Delinquent behavior subscales. Items that are not included in any of the subscales are collected under the heading Other problems. The Total problem score measures the overall behavioral and emotional functioning of the child [44].

The content validity of the competence, adaptive, and problem item scores has been supported by decades of research, and all the findings prove that all items discriminated significantly between demographically matched referred and non-referred children (*p* < 0.01). Additionally, the Internal consistency, the correlation among the CBCL and TRF scales, and the inter-rater correlations for CBCL/TRF scales were good for most indices and subscales [45,46]. Almost all problem scales significantly discriminated between referred and non-referred children [47,48,49].

**Aberrant Behavior Checklist (ABC) (Aman, M.G., Sing. N.N., Stewart, A.W., & Field, C.J., 1985).** The Aberrant Behavior Checklist (ABC) is a standardized problem behavior rating scale that can be completed by a variety of informants in different settings, e.g., parents and teachers. The ABC contains 58 items that form five subscales labeled: Irritability/Agitation/Crying, Lethargy/Social withdrawal, Stereotypic behavior, Hyperactivity/Non-compliance and Inappropriate speech. Each item is scored as 0 (never a problem), 1 (slight problem), 2 (moderately serious problem), or 3 (severe problem).

ABC was shown to have sound psychometric properties with high internal consistency among subscales (mean alpha = 0.91), excellent test-retest reliability (mean r = 0.98), acceptable inter-rater reliability (mean r = 0.63), and moderate correlations with measures of adaptive behavior (mean r = 0.60) [50,51].

### 2.5. Statistical Analysis

Variables were first tested for normality using the Kolmogorov-Smirnov criterion. Quantitative variables were expressed as mean (Standard Deviation) and as median (interquartile range). Qualitative variables were expressed as absolute and relative frequencies. Spearman correlation coefficients were used to explore the association of two continuous variables. In order to adjust for children’s age and gender, partial correlation coefficients were computed. All reported *p*-values are two-tailed. Statistical significance was set at *p* < 0.05, and analyses were conducted using SPSS statistical software (version 22.0).

Given the multiple statistical tests conducted in this study, the risk of Type I error (false positives) due to multiple comparisons is a concern. To mitigate this issue, we applied the Benjamini-Hochberg false discovery rate (FDR) correction (Benjamini & Hochberg, 1995) to adjust *p*-values across multiple comparisons. This method was chosen because it controls for the proportion of false discoveries while maintaining statistical power, making it preferable to more conservative approaches such as the Bonferroni correction, which can be overly restrictive in exploratory studies. Adjusted *p*-values (q-values) are reported for all relevant analyses.

## 3. Results

Data from 63 children, 6 to 12 years old, were collected. Their *characteristics,* as well as their *parents’ characteristics*, information on their medical history, and their condition, are presented in Table 1. Most children were males (81.0%), and their mean age was 9.3 years (SD = 1.9 years). In most cases (79.0%), parents were married and employed (86.2%). Parental history of a mental disease was present in 60.7% of the children, and 36.7% had parents who had used tranquillizers, or psychotropic medication during the last year. Also, 96.7% of the children had visited a specialist, and the most frequent reason for seeking help was children’s unusual behavior. Mean time from children’s symptoms’ onset was 4.6 years (SD = 2.2 years).

*Descriptive statistics* of the study scales are presented in Table 2. Mean total MASK score was 135.81 (SD = 21.82). Also, mean total CBCL score was 53.21 (SD = 26.23) and mean TRF score was 49.94 (SD = 31.56).

*Correlation between MASK and WISC* is presented in Table 3. WISC scores were not significantly associated with either the total MASK score or the Positive Schizotypal Symptoms subscale. However, a greater sum and percentile of Verbal intelligence and a greater sum of Total intelligence were significantly associated with a lower score in the Social/Pragmatic symptoms subscale. After adjusting for children’s gender and age, correlations were no longer significant. It should be noted that after applying the Benjamini-Hochberg correction for multiple comparisons, all correlations dropped to non-significance.

*Correlation between MASK and CBCL scales* is presented in Table 4. Greater Aggressive behavior and more Externalizing problems, according to CBCL, were significantly associated with greater Total MASK score. Also, a greater score in the Withdrawn/depressed subscale was significantly associated with a greater score in the Social/Pragmatic symptoms subscale. CBCL scores were not significantly associated with the Positive schizotypal symptoms subscale. However, after adjusting for children’s gender and age, the correlations of total MASK score with aggressive behavior and externalizing problems score became indicative, r_partial_ = 0.23, *p* = 0.073 and r_partial_ = 0.23, *p* = 0.078, respectively, while the correlation with t-score of externalizing problems became non-significant, *p* > 0.05. Also, after the adjustment, the correlation between the Withdrawn/depressed subscale and the Social/Pragmatic symptoms subscale became non-significant, *p* > 0.05. It should be noted that after applying the Benjamini-Hochberg correction for multiple comparisons, all correlations dropped to non-significance.

*Correlation between MASK and TRF scales* is presented in Table 5. Greater Anxious/depressed scores and more Internalizing problems, according to ΤRF, were significantly associated with greater Total MASK scores. Moreover, greater scores in the Anxious/depressed, Withdrawn/depressed, other problems subscales (e.g., enuresis, desires for the opposite sex, torturing animals, defecating outside the designated toilet area, overweightness, talking too much, etc.) and more Internalizing and total problems were significantly associated with greater scores in the Social/Pragmatic symptoms subscale. Greater Adaptive performance was significantly associated with a greater score on the Positive schizotypal symptoms subscale. After adjusting for children’s gender and age, total MASK score remained significantly correlated with Anxious/depressed score (r_partial_ = 0.28, *p* = 0.028), Internalizing problems score (r_partial_ = 0.26, *p* = 0.043), and internalizing problems t-score (r_partial_ = 0.27, *p* = 0.039). Additionally, after adjusting for children’s gender and age, the Social/Pragmatic symptoms subscale remained significantly correlated with the Anxious/depressed (r_partial_ = 0.29, *p* = 0.023), Withdrawn/depressed (r_partial_ = 0.26, *p* = 0.042), and other problems (r_partial_ = 0.26, *p* = 0.043) subscales and more Internalizing (r_partial_ = 0.31, *p* = 0.018 for raw score and r_partial_ = 0.31, *p* = 0.015 for t-score) and total problems (r_partial_ = 0.25, *p* = 0.050 for raw score and r_partial_ = 0.27, *p* = 0.040 for t-score). The association between Adaptive performance and the Positive schizotypal symptoms subscale, after adjusting for children’s gender and age, became indicative, r_partial_ = 0.24, *p* = 0.064. After applying the Benjamini-Hochberg correction for multiple comparisons, all correlations dropped to non-significance.

*Correlation between MASK and ABC scales* is presented in Table 6. Greater teacher’s score on the Irritability and Inappropriate speech subscales were significantly associated with greater total MASK scores. Social/Pragmatic symptoms were not significantly correlated with ABC subscales. Furthermore, a greater teacher’s score on the *Inappropriate speech subscale* was significantly associated with a greater score on the Positive schizotypal symptoms subscale. Parents’ ABC scores were not associated with MASK scores. After adjusting for children’s gender and age, total MASK score remained significantly associated with the Inappropriate speech subscale, r_partial_ = 0.35, *p* = 0.007, while its association with the Irritability subscale, became indicative, r_partial_ = 0.24, *p* = 0.067. Moreover, after adjusting for children’s gender and age, the correlation between the Positive schizotypal symptoms subscale and the Inappropriate speech subscale remained significant, r_partial_ = 0.27, *p* = 0.040. It should be noted that after applying the Benjamini-Hochberg correction for multiple comparisons, all correlations dropped to non-significance. It should be noted that after applying the Benjamini-Hochberg correction for multiple comparisons, all correlations dropped to non-significance.

## 4. Discussion

The present study aimed to explore how schizotypal traits impact the social, emotional, and behavioral functioning of children with Autism Spectrum Disorder.

One of the key challenges in studying the co-occurrence of Autism Spectrum Disorder (ASD) and schizotypal traits is the lack of a universally accepted measurement tool that captures schizotypy in childhood. The Melbourne Assessment of Schizotypy in Kids (MASK) was developed as a semi-structured assessment tool to capture schizotypal traits in children [8], yet its conceptual alignment with DSM-5 schizotypy remains an area of debate. Specifically, the Social/Pragmatic factor within MASK includes features commonly associated with ASD, raising concerns about construct overlap. While the Positive Schizotypal factor is intended to differentiate ASD from schizotypy, studies have shown that this factor does not always correlate strongly with established measures of developmental psychopathology [8].

Given these limitations, the present study adopts a dimensional approach rather than a categorical framework and acknowledges the need for further refinement in assessing schizotypal traits in children with ASD. Future research should consider alternative schizotypy measures, particularly those that align more closely with DSM-5 conceptualizations.

In the studied group of 63 children, most children were males (81.0%), which is in agreement with the ratio of boys to girls as recorded in the literature (see systematic review and meta-analysis by Loomes et al. [52]). By observing the characteristics of the parents, it seems that a large percentage (60.7%) of the children had a parental or family history of a mental disease, while 36.7% had parents who had used tranquillizers or psychotropic medication during the last year. These findings reflect the significant impact a child with autism has on the family, including elevated levels of psychiatric symptoms among parents [53,54,55].

In the present study, the children’s behavior was recorded by their parents and teachers. An interesting finding of the present study is that there is a difference in the way children’s behaviors are recorded by parents and teachers in the school setting.

Overall, from the correlations between the above clinical tool MASK and the Checklists the results report that (1) *Total MASK score* is significantly correlated with Internalizing problems and Anxious/depressed scores, according to TRF, (2) *MASK Social/Pragmatic symptoms score* is significantly correlated with Internalizing problems, Total problems, Anxious/Depressed, Withdrawn/Depressed, and Other problems scores, according to the TRF, (3) *MASK Positive schizotypal score* and *Total MASK score* are significantly associated with Inappropriate speech score, according to teacher’s ABC scales. While several associations between MASK scores and behavioral/emotional indices appeared statistically significant prior to correction, none of these remained significant after applying the Benjamini-Hochberg correction for multiple comparisons. As such, the current findings must be considered exploratory and hypothesis-generating rather than confirmatory. The discussion of patterns and trends below is provided to guide future research and not to imply definitive or clinically actionable relationships.

In the literature there are studies that document gaps in the way parents and teachers understand the child’s behavior [56]. However, there are not sufficient studies about behavioral problems in students with ASD in relation to inter-rater agreement in multi-informant reports such as parents and teachers using the Child Behaviour Checklist (CBCL) and Teacher Rating Form (TRF) [57]. Kohler [58] investigated parent versus teacher reports on the behavior of neurotypical children and came to the conclusion that agreement between parents and teachers was poor, with parents reporting more problematic behavior compared to teachers. Zope [59] investigated parent versus teacher reports on the behavior of pediatric cancer survivors and recorded that parents had higher ratings of child-internalizing problems but lower ratings of overall social skills than teacher ratings. Ιn the same study, parent-teacher agreement was higher for reports of externalizing symptoms. Roussos et al. [45], in the context of the standardization process for the CBCL and TRF questionnaires for Greek children aged 6–12, examined the correlations between parents’ and teachers’ ratings of problem behaviors for boys and girls. The study revealed that, for boys, parents and teachers exhibited the highest correlations on the Externalizing dimension. Specifically, the Aggressive Behavior scale demonstrated the strongest correlation between CBCL and TRF among the narrow-band scales, followed closely by the Attention Problems scale. For girls, a high correlation was observed only on the Attention Problems scale.

### 4.1. MASK & ΤRF Relation

A significant correlation was found, after adjusting for children’s gender and age, between the *Social/Pragmatic symptoms subscale* and Internalizing problems, mainly regarding syndromes: Anxious/depressed, Withdrawn/depressed and Other problems. Additionally, the *Total MASK score* also remained significantly correlated with Internalizing problems and Anxious/depressed syndrome. However, it is important to note that none of these associations retained statistical significance after applying the Benjamini-Hochberg correction for multiple comparisons.

This suggests that children with higher schizotypal traits, as measured by the MASK, may have problems that are described by the following two TRF subscales. According to the Anxious/depressed subscale, the child cries a lot, has fears (certain animals, situations, places), is afraid of going to school, feels the need to be perfect, feels unloved, feels worthless or inferior, nervous, fearful, or anxious, feels overly guilty, is easily embarrassed, and/or worries. According to the Withdrawn/depressed subscale, there is very little for the child to enjoy, would rather be alone than with others, refuses to talk, keeps things to themself, is too timid, is underactive (slow moving or lacks energy), is sad (depressed) and/or is withdrawn (does not get involved with others). However, given the lack of significance after correction, these observations should be interpreted cautiously and warrant further investigation in larger samples.

The role of social pragmatic difficulties in the development of internalizing symptoms in children with ASD appears to be significant. Pragmatic language impairments can cause a child to misinterpret social cues and interactions. These challenges in social understanding may lead to feelings of insecurity, which, in turn, are likely to contribute to heightened anxiety [60,61].

According to the literature, ‘internalizing’ behaviors like withdrawal, anxiety, and depression are common in individuals with ASD [62]. Depression and Anxiety are among the most frequently co-occurring disorders in individuals with ASD, especially in those with higher-functioning autism who are able to articulate their challenges [63,64]. 40% of youth with ASD are estimated to experience a comorbid anxiety disorder, and 10% meet criteria for a mood disorder [65,66]. On the other hand, internalizing disorders significantly affect quality of life by worsening ASD symptoms and disrupting social functioning [67,68]. In the past decade, psychosocial interventions and treatments, such as Cognitive Behavioral Therapy (CBT) adapted for the ASD population, have been developed [65]. To reiterate, the present results do not support definitive claims about the link between schizotypal traits and internalizing symptoms but rather highlight an area that warrants further exploration. Larger studies with pre-registered hypotheses and sufficient statistical power are needed to validate or refute these preliminary observations.

### 4.2. MASK & ABC Relation

After adjusting for children’s gender and age, a significant correlation was found between the *Positive schizotypal symptoms subscale* and the *Inappropriate speech subscale* of the teacher’s ABC. Additionally, the *Total MASK score* remained significantly correlated with the *Inappropriate speech subscale*. However, none of these associations retained statistical significance after applying the Benjamini-Hochberg correction for multiple comparisons. These preliminary trends may suggest that children with higher schizotypal traits, as measured by the MASK, exhibit inappropriate speech compared to their classmates in the school setting and in a given situation.

The presence of schizotypal traits appears to further impact the social and academic development of school-age children with ASD, with social and pragmatic difficulties being more evident in the school setting. This may be because, in the school environment, children are directly compared to their peers in terms of both academic performance and functional abilities within the same context and timeframe.

Schizotypal Personality Disorder (SPD) is classified within the schizophrenia spectrum in both the DSM-5 and the ICD-11 as part of a continuum of psychotic disorders. It is already known from clinical experience and research that communication disturbances are a common symptom of schizophrenia and related to disturbance of thought process [69,70]. Several different types of communication disturbances have been identified in the speech of schizophrenia patients and in people at risk for schizophrenia [71].

SPD is characterized by thought disorder, paranoia, social anxiety, derealization, transient psychosis, and unconventional beliefs. Persons with SPD frequently interpret situations as strange or having unusual meanings, with paranormal and superstitious beliefs being common. They may also display socially unexpected behaviors, such as odd or eccentric dress. In conversations, they might react unusually by not responding, talking to themselves, or exhibiting peculiar speech mannerisms [72]. In the present study, we reached similar conclusions, as higher positive schizotypal symptoms were found to correlate significantly with inappropriate speech observed in the school setting.

Similar to the MASK and ΤRF relation, while the associations observed in the present study initially aligned with these clinical characteristics, they should not be interpreted as confirmatory. Given that all associations lost statistical significance after correction, the findings remain speculative and should be viewed as exploratory indicators for future study.

Referring to the historical construct of schizotypy as introduced by Meehl in 1964, it is evident that thought disturbances (Meehl named them as *cognitive slippage)* were the most dependable indictor of schizotypy, and they were a primary manifestation of a schizophrenia diathesis [73]. Snitz et al. [74], in a meta-analytic review, provided evidence that cognitive deficits are present in the small- to medium-effect-size range in unaffected adult first-degree relatives of schizophrenia patients. Reliable group differences were still found on tasks of language. These findings are highly significant, as cognitive deficits may serve as indicators of genetic susceptibility to schizophrenia and are considered potential endophenotypes for the illness.

The findings of the current study regarding the impact of varying degrees of schizotypal traits on the social, emotional, and behavioral functioning of children with ASD contribute to the existing literature. Although some associations were initially significant, none withstood correction for multiple comparisons. As such, these findings must be interpreted as hypothesis-generating rather than confirmatory. Nevertheless, the observed patterns echo existing literature and clinical observations, underscoring the importance of further research using larger samples, more refined instruments, and pre-registered hypotheses to rigorously explore these relationships.

### 4.3. Limitations of the Study

A significant limitation of the present study is that it did not include data on neurotypical children and children with Broader Autism Phenotype (BAP) (*autistic traits*) or other diagnoses. The study of distinct control groups with neurotypical children with various diagnoses, such as ASD only, schizotypy only, and co-occurring ASD and schizotypy, would have allowed for a more rigorous examination of how autistic and schizotypal traits correlate and interact and how this interaction is captured in behavior, emotion, and functioning. The lack of such groups limits the interpretability of the observed associations and prevents stronger inferences about specificity or additive effects. Future studies would greatly benefit from the inclusion of well-matched comparison groups to strengthen internal validity and clarify differential patterns of social-emotional impairment.

One important limitation of this study is the use of the Wechsler Intelligence Scale for Children-Third Edition (WISC-III; Wechsler, 1992). Although this version remains validated for the Greek population (Georgas et al., 1997 [75]), at the time of data collection, more recent editions had not yet been fully standardized in Greece for clinical or research use. However, the use of an older version may limit the generalizability of the cognitive findings to international contexts, where newer versions (e.g., WISC-V) are now the standard and may include updated norms, broader index scores, and improved psychometric properties. Future studies should consider utilizing updated intelligence measures to enhance the validity and generalizability of their findings.

Another limitation is that the study’s relatively small sample size may restrict its ability to detect subtle effects or interactions between multiple and complex variables. However, this limitation can ultimately be overcome, as the study was originally designed based on the dimensional approach model, where each individual receives a score on a continuous scale of psychological distress without a cut-off point to define a boundary between those with and without a presumed illness.

An additional limitation of the present study is the potential inflation of significance levels due to multiple comparisons. Although our analyses were hypothesis-driven and guided by prior literature, applying Benjamini-Hochberg correction led to most correlations falling below the significance threshold. No firm conclusions should be drawn based on uncorrected results, and any interpretive discussion has been framed to reflect the exploratory nature of the study. While this underscores the importance of cautious interpretation, the observed patterns remain consistent with previous research. Future studies should replicate these trends in larger samples with adequate power and prespecified hypotheses to enable more definitive conclusions.

## 5. Conclusions

In conclusion, our preliminary findings highlight the need for further research regarding the phenomenological, genetic, imaging, and environmental overlaps of schizophrenia and autism spectra. Research shows that the large clinical heterogeneity of the spectrum suggests multiple underlying causes with different developmental manifestations.

Τhere is a need to design longitudinal studies to clarify the developmental trajectories of autistic and schizotypal traits. It is important to study the development of children who exhibit odd and/or intense imagination and confusion between reality and fantasy and to gain a deeper understanding of these characteristics in order to identify those who may develop psychosis as they grow older.

Distinguishing children with autism who also exhibit schizotypal characteristics presents both diagnostic and therapeutic challenges. The ultimate goal is to determine the “nature” of the symptoms in this group of children. They might be classified as one of the following: an “endophenotype” within the autism spectrum, a “comorbid condition” involving both autism and schizophrenia, or a “phenotypic variant” of very early-onset schizophrenia (VEOS).

It is essential to develop accurate standardized psychometric tools for the co-assessment of autism and schizophrenia traits and to design targeted personalized interventions to prevent emotional distress and the development of additional dysfunctional behaviors. The goal is to ensure better functionality and quality of life for children and their families.

## Figures and Tables

**Table 1:** Sample’s characteristics.

		Ν (%)
Child’s characteristics		
Child’s gender		
	Males	51 (81.0)
	Females	12 (19.0)
Child’s age, mean (SD)		9.3 (1.9)
Parallel educational support		22 (43.1)
Parental characteristics		
Father’s age, mean (SD)		45.2 (7.1)
Mother’s age, mean (SD)		42.0 (6.6)
Family status		
	Unmarried	5 (8.1)
	Married	49 (79.0)
	Separated	5 (8.1)
	Divorced	3 (4.8)
Employed		50 (86.2)
Family history		
Parental or family history of a mental disease		34 (60.7)
Parental or family history of a serious disease		11 (18.3)
History of drug/alcohol abuse		3 (5.3)
Parental use of tranquillizers or psychotropic medication during last year		22 (36.7)
Child’s use of tranquillizers or psychotropic medication during last year		3 (4.8)
Information on child’s condition		
Visited a specialist		59 (96.7)
Reasons for seeking help		
	Talking delay	29 (46)
	Weird behaviour	40 (63.5)
	Anxiety	14 (22.2)
	Medical problem	2 (3.2)
	Problems associated with relationships or family	1 (1.6)
	Information on diagnosis	4 (6.3)
	Prescription	5 (7.9)
	Assessment	4 (6.3)
Years since symptoms’ onset, mean (SD)		4.6 (2.2)
Years knowing childs’ developmental disorders, mean (SD)		3.9 (2.6)

**Table 2:** Descriptive statistics of under study scales.

		Minimum	Maximum	Mean (SD)	Median (IQR)
Mask					
	Total score	99	192	135.81 (21.82)	133 (118–147)
	Social/Pragmatic symptoms	61	110	83.44 (10.28)	84 (76–91)
	Positive schizotypal symptoms	33	94	53.25 (15.56)	48 (42–58)
WISC					
Verbal intelligence	Subtest Scores	15	77	50.18 (15.45)	50 (40.5–62)
	Verbal intelligence Quotient	58	150	104.89 (19.62)	100 (94–121)
	Percentile	0.3	125	57.17 (36.46)	57 (21–94)
Practical intelligence	Subtest Scores	8	84	45.04 (15.96)	47 (32.5–55.5)
	Performance intelligence Quotient	52	148	98.88 (19.62)	101 (86–111)
	Percentile	0.1	100	50.68 (34.57)	58 (16–79)
Total intelligence	Subtest Scores	35	161	95.21 (28.2)	99.5 (77–118)
	Full scale intelligence Quotient	71	148	101.83 (18.4)	100.5 (85.5–115)
	Percentile	0	99.9	51.55 (36.34)	54 (14–87)
CBCL					
	Activities	0	14.3	8.23 (3)	8 (6–11)
	Social/Pragmatic symptoms	0	11	5.13 (2.52)	5.6 (3–7)
	Academic performance	0	8	3.09 (1.65)	3 (2–4)
	Total competence	6	28.1	16.44 (5.14)	16 (13–20.5)
	Total competence t-score	17	56	32.29 (9.4)	29 (25–39)
	Anxious/depressed	0	19	8.56 (4.27)	8 (6–11)
	Withdrawn/depressed	0	13	4.44 (2.86)	4 (2–6)
	Somatic complaints	0	9	1.97 (2.44)	1 (0–3)
	Social problems	0	16	6.9 (4.2)	7 (3–10)
	Thought problems	0	18	5.83 (3.84)	5 (3–9)
	Attention problems	0	18	8.06 (4.31)	8 (5–11)
	Rule-breaking behaviour	0	20	3.43 (3.6)	2 (1–5)
	Aggressive behaviour	0	28	8.98 (6.3)	8 (3–13)
	Other problems	0	13	5.03 (3.28)	5 (2–8)
	Internalizing problems	1	32	14.97 (7.6)	15 (10–19)
	Internalizing problems t-score	39	78	64.48 (8.59)	66 (61–70)
	Externalizing problems	0	48	12.41 (9.38)	10 (6–18)
	Externalizing problems t-score	33	83	58.49 (9.97)	57 (51–66)
	Total problems	6	118	53.21 (26.23)	53 (31–71)
	Total problems t-score	39	79	63.49 (9.49)	66 (56–71)
TRF					
	Academic performance	0	5	2.66 (1.09)	2.8 (2–3.2)
	Adaptive performance	7	26	15.39 (4.17)	15 (13–18)
	Anxious/depressed	0	26	7.34 (5.62)	6 (4–9)
	Withdrawn/depressed	0	15	4.73 (3.47)	4 (2–7)
	Somatic complaints	0	13	0.84 (2.23)	0 (0–1)
	Social problems	0	16	5.23 (3.7)	4 (3–8)
	Thought problems	0	17	4.1 (3.93)	4 (1–7)
	Inattention	0	22	10.02 (5.91)	10.5 (4–14)
	Hyperactivity-Impulsivity	0	21	6.42 (5.75)	5.5 (1–9)
	Attention problems	0	43	16.44 (10.51)	17 (7–24)
	Rule-breaking behaviour	0	15	2.34 (2.89)	2 (0–3)
	Aggressive behaviour	0	30	7.69 (7.56)	5 (2–14)
	Other problems	0	12	1.24 (1.82)	1 (0–2)
	Internalizing problems	0	49	12.9 (9.19)	11 (6–18)
	Internalizing problems t-score	37	91	61.77 (9.98)	62 (56–68)
	Externalizing problems	0	40	10.03 (9.91)	7 (3–15)
	Externalizing problems t-score	43	82	60.89 (8.91)	61 (57–67)
	Total problems	0	177	49.94 (31.56)	50 (26–69)
	Total problems t-score	33	93	63 (9.56)	65.5 (58–68)
ABC					
Parents	Irritability	0	37	9.74 (7.97)	8 (4–14)
	Lethargy/Withdrawal	0	34	11.69 (9.4)	8 (4–18)
	Stereottypy	0	17	4.48 (4.26)	4 (1–7)
	Hyperactivity	0	48	13.76 (11.69)	11 (6–20)
	Inappropriate speech	0	11	2.66 (2.54)	3 (0–4)
Teachers	Irritability	0	28	8.69 (7.46)	6.5 (3–13)
	Lethargy/Withdrawal	0	40	12.4 (8.95)	11 (6–17)
	Stereottypy	0	18	4.55 (4.62)	3.5 (0–7)
	Hyperactivity	0	37	11.61 (8.65)	10 (6–18)
	Inappropriate speech	0	12	2.97 (2.82)	2 (1–5)

**Table 3:** Spearman’s correlation coefficients between MASK and WISC scales.

WISC		Total MASK Score		Social/Pragmatic Symptoms		Positive Schizotypal Symptoms	
		rho	*p*	rho	*p*	rho	*p*
Verbal intelligence	Subtest Scores	−0.26	0.057	−0.27	0.048	−0.20	0.138
	Verbal intelligence Quotient	−0.19	0.158	−0.21	0.111	−0.11	0.400
	Percentile	−0.26	0.118	−0.35	0.030	−0.14	0.393
Practical intelligence	Subtest Scores	−0.08	0.547	−0.20	0.147	−0.04	0.759
	Performance intelligence Quotient	−0.05	0.712	−0.09	0.499	−0.05	0.739
	Percentile	−0.27	0.104	−0.29	0.087	−0.32	0.054
Total intelligence	Subtest Scores	−0.21	0.112	−0.27	0.042	−0.17	0.221
	Full scale intelligence Quotient	−0.20	0.128	−0.22	0.087	−0.14	0.282
	Percentile	−0.18	0.276	−0.25	0.123	−0.12	0.462

**Table 4:** Spearman’s correlation coefficients between MASK and CBCL scales.

	Total MASK Score		Social/Pragmatic Symptoms		Positive Schizotypal Symptoms	
CBCL	rho	*p*	rho	*p*	rho	*p*
Activities	0.11	0.381	0.08	0.537	0.17	0.183
Social/Pragmatic symptoms	0.06	0.626	0.14	0.283	0.04	0.732
Academic performance	0.02	0.882	0.03	0.821	0.06	0.616
Total competence	0.08	0.513	0.10	0.425	0.13	0.295
Total competence t-score	0.08	0.522	0.10	0.426	0.12	0.341
Anxious/depressed	0.23	0.075	0.18	0.170	0.18	0.154
Withdrawn/depressed	0.23	0.075	0.25	0.050	0.12	0.329
Somatic complaints	−0.01	0.948	0.15	0.233	−0.10	0.451
Social problems	0.14	0.288	−0.01	0.945	0.17	0.187
Thought problems	0.18	0.158	0.16	0.209	0.07	0.585
Attention problems	0.23	0.074	0.07	0.586	0.19	0.127
Rule-breaking behaviour	0.15	0.245	−0.01	0.927	0.15	0.234
Aggressive behaviour	0.25	0.048	0.10	0.458	0.23	0.073
Other problems	0.10	0.450	0.08	0.544	0.10	0.455
Internalizing problems	0.22	0.086	0.22	0.091	0.14	0.257
Internalizing problems t-score	0.20	0.119	0.20	0.109	0.13	0.297
Externalizing problems	0.25	0.050	0.09	0.489	0.22	0.091
Externalizing problems t-score	0.25	0.046	0.08	0.532	0.24	0.061
Total problems	0.21	0.094	0.09	0.466	0.18	0.160
Total problems t-score	0.22	0.079	0.10	0.445	0.19	0.136

**Table 5:** Spearman’s correlation coefficients between MASK and TRF scales.

	Total MASK Score		Social/Pragmatic Symptoms		Positive Schizotypal Symptoms	
TRF	rho	*p*	rho	*p*	rho	*p*
Academic performance	0.17	0.181	0.09	0.493	0.17	0.199
Adaptive performance	0.18	0.173	0.02	0.886	0.28	0.028
Anxious/depressed	0.28	0.027	0.31	0.014	0.22	0.091
Withdrawn/depressed	0.13	0.310	0.26	0.044	−0.04	0.740
Somatic complaints	0.13	0.322	0.14	0.275	0.04	0.755
Social problems	0.11	0.374	0.13	0.315	0.11	0.381
Thought problems	0.13	0.317	0.24	0.060	0.03	0.797
Inattention	0.08	0.520	0.11	0.396	0.00	0.969
Hyperactivity-Impulsivity	0.16	0.221	0.21	0.100	0.03	0.804
Attention problems	0.15	0.239	0.19	0.130	0.04	0.782
Rule-breaking behaviour	0.08	0.534	0.00	0.985	0.05	0.682
Aggressive behaviour	0.13	0.331	0.13	0.324	0.06	0.671
Other problems	0.13	0.305	0.26	0.044	0.03	0.827
Internalizing problems	0.26	0.045	0.32	0.013	0.13	0.304
Internalizing problems t-score	0.25	0.046	0.32	0.010	0.12	0.337
Externalizing problems	0.15	0.255	0.12	0.334	0.08	0.542
Externalizing problems t-score	0.15	0.256	0.12	0.350	0.08	0.513
Total problems	0.20	0.124	0.27	0.032	0.07	0.579
Total problems t-score	0.19	0.130	0.29	0.024	0.06	0.655

**Table 6:** Spearman’s correlation coefficients between MASK and ABC scales.

		Total MASK Score		Social/Pragmatic Symptoms		Positive Schizotypal Symptoms	
ABC		rho	*p*	rho	*p*	rho	*p*
Parents	Irritability	0.09	0.488	−0.04	0.757	0.10	0.437
	Lethargy/Withdrawal	0.05	0.698	−0.03	0.819	0.03	0.796
	Stereottypy	−0.04	0.747	−0.06	0.646	−0.03	0.798
	Hyperactivity	−0.01	0.919	−0.12	0.372	0.02	0.899
	Inappropriate speech	0.22	0.085	0.08	0.531	0.23	0.076
Teachers	Irritability	0.25	0.049	0.19	0.139	0.15	0.250
	Lethargy/Withdrawal	0.01	0.909	0.09	0.472	−0.09	0.488
	Stereottypy	0.14	0.284	0.16	0.228	0.02	0.855
	Hyperactivity	0.08	0.526	0.07	0.592	−0.02	0.883
	Inappropriate speech	0.34	0.007	0.21	0.108	0.25	0.050

## Data Availability

The original contributions presented in this study are included in the article. Further inquiries can be directed to the corresponding author.
